# Performance of women with fibromyalgia in walking up stairs while carrying a load

**DOI:** 10.7717/peerj.1656

**Published:** 2016-02-01

**Authors:** Daniel Collado-Mateo, José C. Adsuar, Pedro R. Olivares, Francisco J. Dominguez-Muñoz, Cristina Maestre-Cascales, Narcis Gusi

**Affiliations:** 1 Faculty of Sport Science, University of Extremadura, Cáceres, Spain; 2 Instituto de Actividad Física y Salud, Universidad Autonoma de Chile, Talca, Chile; 3 Instituto Superior de Educación Física, Universidad de la República, Uruguay

**Keywords:** Fibromyalgia, Postural balance, Women, Daily living, Stair ascent, Motor control

## Abstract

**Background.** Fibromyalgia is a chronic disease characterized by widespread pain and other associated symptoms. It has a relevant impact on physical fitness and the ability to perform daily living tasks. The objective of the study was to analyze the step-by-step-performance and the trunk tilt of women with fibromyalgia in the 10-step stair climbing test compared with healthy controls.

**Methods.** A cross-sectional study was carried out. Twelve women suffering from fibromyalgia and eight healthy controls were recruited from a local association. Participants were asked to climb 10 stairs without carrying a load and 10 stairs carrying a load of 5 kg in each hand. Mediolateral trunk tilt was assessed using the “Functional Assessment of Biomechanics (FAB)” wireless motion capture device, and the time between steps was assessed via weight-bearing insoles.

**Results.** Trunk tilt in the stair-climbing task carrying a load was significantly higher in women with fibromyalgia when compared to the healthy controls (2.31 (0.63) *vs.* 1.69 (0.51) respectively). The effect of carrying a load was significantly higher for women with fibromyalgia compared with healthy controls at the intermediate and final part of the task.

**Discussion.** Trunk tilt during stair climbing while carrying a load was higher in women with FM, which could increase the risk of falling. Additionally, women with FM experienced a higher pace slowdown as a consequence of the load, which supports the need of including specific strength and resistance training to physical therapies for this population.

## Introduction

Fibromyalgia (FM) is a chronic disease of unknown aetiology. It is characterized by widespread pain and several associated symptoms, such as fatigue, non-restorative sleep, mood alterations, and poor health-related quality of life (HRQoL) (Segura-Jimenez et al., 2014; Wolfe, 2015). In Spain, approximately 4.2% of the women suffer from this disorder, whereas only 0.2% of the men are affected (Mas et al., 2008).

Physical fitness of women with FM has been previously studied. Among the physical impairments of FM patients, low levels of strength, endurance, flexibility, and poor balance are extremely relevant (Aparicio et al., 2015; Muto et al., 2015). In fact, it is suggested that FM may have a larger impact on physical health rather than on psychological health (Segura-Jimenez et al., 2015). In this regard, physical fitness has been associated with a number of FM symptoms, such as pain (Carbonell-Baeza et al., 2011), HRQoL (Latorre-Roman et al., 2014), anxiety and depression (Sener et al., 2013).

There is a growing interest in quantifying the impact which FM has on the performance of ADLs. The work of Huijnen et al. (2015) demonstrated that FM may have a significant impact on the performance and perception of ADLs. Specifically, the capacity to ascend and descend stairs with and without a load can be an important and physically challenging ADL of women with FM and can be potential source of injury due to falls. In this regard, a recent study also reported that women with FM have more than three times more falls than healthy controls during the period of one year (1.45 and 0.40 respectively). These women also reported higher fear of falling and lower levels of objective and perceived balance ability (Collado-Mateo et al., 2015). The circumstances of falls among women with FM were investigated by Rutledge et al. (2013). Most falls occurred at home (67%), during the day (89%) and while walking (44%) or on stairs (39%). Additionally, most of the falls on stairs occurred during stair ascent. Therefore, stair climbing could be a focus for healthcare providers.

Climbing stairs is an extremely important ADL for women with FM. The Fibromyalgia Impact Questionnaire (FIQ) (Bennett, 2005) and its Revised version (FIQ-R) (Bennett et al., 2009b) include an item asking whether patients were able to climb stairs and the difficulty that they experienced while climbing stairs, respectively. Climbing stairs can be measured through a physical test. Previous studies utilized a physical test in which patients with FM had to climb ten stairs as fast as possible (Valkeinen et al., 2004). Most of the studies utilized this method to evaluate the effects of a training program proving that physical exercise could prevent the progressive loss of performance climbing stairs weightless or carrying loads (Tomas-Carus et al., 2008). Two studies utilized it to compare the performance of FM patients and healthy controls (Huijnen et al., 2015; Valkeinen et al., 2008). These two studies concluded that patients with FM needed more time to complete the stair-climbing task. However, to our knowledge, no studies have explored the step-by-step-performance of FM patients in a stair-climbing task.

It is a well-known fact that stair-climbing is a common ADL, but this task is often performed while carrying loads. In fact, the FIQ-R included one item asking about how much FM influenced the difficulty to “lift and carry a bag full of groceries.” Previous studies with healthy people investigated the effects of carrying a load while ascending stairs or walking (Hall et al., 2013; Tseng & Liu, 2011). Among load-carrying methods, hand-held loads seems to cause major effects on the heart-rate, perceived exertion, and postural control (Tseng & Liu, 2011). Therefore, the most common load-carrying method in FM women according with FIQ-R is also the most physically demanding method. However, to our knowledge no studies have investigated the impact of carrying a load on the motor pattern of stair ascent in FM patients.

FM is related to some neurological symptoms that may cause poor balance and coordination, i.e., vision problems, ringing ears, vertigo, dizziness, etc. (Watson et al., 2009). Maintaining postural control and balance while climbing stairs is crucial to avoid falls. In this regard and to the best of our knowledge, no studies have evaluated the stability (through trunk or pelvic tilt, displacement or velocity of the center of pressure, or other measure) of women with FM in the stair-climbing task.

The aim of the current paper was to analyze the step-by-step-performance and the trunk tilt of women with FM in the 10-step stair-climbing test compared with healthy controls.

## Materials & Methods

### Participants

A total of 12 women with FM and eight healthy controls underwent the stair ascent tasks. All participants were recruited from a local association. Some inclusion and exclusion criteria were set: (a) be able to climb 10 stairs while carrying a load of 5 kg in each hand (participants were excluded if they tried but were not able), (b) be aged between 18 and 75 years. Additionally, women with FM were included if they had been diagnosed with FM by a rheumatologist accordingly with the criteria of the American College of Rheumatology (Wolfe et al., 1990). From a sample of 24 women, three women with FM and one healthy woman were excluded because they were not able to climb 10 stairs while carrying the load. Finally, 20 women provided written informed consent accordingly to the updated Declaration of Helsinki. The Bioethical Committee of the University of Extremadura (Spain) approved this study to be conducted (Registration number: 113/2014).

### Procedure

In first place, anthropometric measurements were collected and entered in the software of the motion capture device. Participants were also measured and weighed using Tanita BC-418 MA body composition analyzer. Sensors of the motion capture device were placed across standard body locations (Rahimi et al., 2011). Once the sensors were placed and the device calibrated, participants climbed the 10 stairs without carrying a load, they rested for 3 min and then they repeated the task, but this time carrying a load of 5 kg in each hand. The riser height of each stair was 17 cm, and its tread depth was 28 cm for safety reasons (Jackson & Cohen, 1995; Tseng & Liu, 2011). Finally, women with FM completed the revised version of the FM Impact Questionnaire (FIQ-R).

### Materials and measures

Kinematic data was assessed using the Functional Assessment of Biomechanics ™ (FAB) System (Biosyn Systems Inc., Surrey, BC, Canada). It is wireless motions capture system that consists of 13 sensors, which contain accelerometers, magnetometers, and gyroscopes. Data was collected at 100 Hz. The FAB assesses angles, positions, velocities, and accelerations of body segments. The FAB also collects foot sole pressure using weight-bearing insoles. This novel device was previously used to evaluate whole body mobility in Parkinson’s disease (Rahimi et al., 2014; Rahimi et al., 2011), and velocity and range of motion in cyclists (Corbett, Peer & Ridgel, 2013).

In the stair-climbing tasks, the mean of the absolute value of the medio-lateral sway of the trunk, that is, trunk tilt was assessed. This parameter is directly assessed by the FAB. Time spent between stairs was also calculated using data from weight bearing sensors. First and last stairs were excluded from the analysis as they cannot be compared with the rest because they start or finish with both feet on the ground.

In order to characterize the sample, the impact of FM was assessed using both the FM Impact Questionnaire (FIQ) and the Revised FM Impact Questionnaire (FIQ-R). FIQ is a 10-item, self-administered instrument (Bennett, 2005). The items consist on physical impairment, feel good, work missed, do job, pain, fatigue, rested, stiffness, anxiety, and depression. A consensus version for Spanish population was developed by Esteve-Vives et al. (2007). The FIQ was revised in 2009, originating a new questionnaire called FIQ-R (Bennett et al., 2009b). The validation of the Spanish version of FIQ-R was developed by Salgueiro et al. (2013).

### Statistical analysis

Characteristics of women with FM and healthy controls were compared using the unpaired Student’s *t* test. The Kolmogorov Smirnov and Shapiro tests were used to determine normality. Normal distribution was assumed when the *p*-value was higher than .05. Differences between groups in trunk tilt were evaluated by comparing mean and median trunk tilt in the task of carrying a load and in the task without carrying a load using the independent sample Student’s *t*-test or Mann–Whitney U test, for parametric and non-parametric variables respectively. The eight analyzed stairs were grouped into: (a) initial stairs (stairs 1, 2 and 3); (b) intermediate stairs (stairs 4 and 5); and (c) final stairs (stairs 6, 7 and 8). A load effect was calculated in the step-by-step analysis. It was defined as: Load effect (%) = (step duration while carrying a load-step duration without carrying a load)/step duration without carrying a load ∗ 100. It represents the percentage rate for the increase as consequence of carrying a load. Load effect coefficient was compared between the two groups using the independent sample Student’s *t*-test or Mann–Whitney *U* test, where appropriate. Cohen’s *d* was calculated as the difference between means divided by the pooled standard deviation appropriately weighed for groups with non-equal sample sizes (Cohen, 1988). An effect size <0.5 was considered small, between 0.5 and 0.8 was considered medium, and >0.8 was considered large (Cohen, 1988).

## Results

Main characteristics of participants are depicted in Table 1. No significant between-group differences were observed in age, height, weight, muscular mass, fat mass, and body mass index (BMI).

**Table 1:** Main characteristics of fibromyalgia women and healthy women.

	Fibromyalgia women (*n* = 12)	Healthy women (*n* = 8)	*p*-value^*^
Age (years)	5.25 (9.71)	59.00 (13.72)	.741
Height (cm)	156.67 (3.03)	157.75 (4.13)	.506
Body mass (kg)	71.31 (9.24)	70.05 (7.43)	.752
Muscular mass (%)	57.26 (4.53)	57.65 (4.00)	.845
Fat mass (%)	39.71 (4.81)	39.28 (1.54)	.840
Body mass index (kg/m^2^)	29.15 (4.43)	28.19 (3.39)	.610
Years since fibromyalgia symptoms started	17.42 (10.78)	NA	NA
Years since fibromyalgia diagnosis	8.75 (2.63)	NA	NA
FIQ-R function domain	13.39 (6.35)	NA	NA
FIQ-R overall Impact domain	5.83 (4.22)	NA	NA
FIQ-R t symptom domain	30.29 (8.13)	NA	NA
FIQ-R total score	49.51 (14.80)	NA	NA
FIQ total score	50.44 (11.62)	NA	NA
FIQ pain (from 0 to 10)	5.08 (3.03)	NA	NA

**Notes.**

Values expressed as mean (SD).

NA, Not applicable.

* *p*-value of the Student’s *t* test.

As can be seen in Table 2, mean trunk tilt in the stair-climbing task carrying a load was significantly higher in women with FM compared to healthy controls (2.31 (0.63) *vs.* 1.69 (0.51) from midline respectively). Similar results were observed for the median trunk tilt. There were not differences in trunk tilt in the same task without carrying a load. The effect size was large (>0.8) when carrying a load and small without carrying a load.

**Table 2:** Load effect in women with and without fibromyalgia.

	Shapiro Wilk (*p*-value)	Fibromyalgia women (*n* = 12)	Healthy women (*n* = 8)	*p*-value^*^	Effect size (Cohen’s *d*)
Trunk tilt					
Mean tilt carrying a load (°)	.342	2.31 (0.63)	1.69 (0.51)	.034	1.11^c^
Mean tilt without carrying a load (°)	.653	2.16 (0.60)	2.05 (0.79)	.712	0.17^a^
Median tilt carrying a load (°)	.025	2.22 (0.78)	1.43 (0.47)	.007	1.23^c^
Median tilt without carrying a load (°)	.868	1.98 (0.57)	1.80 (1.03)	.624	0.24^a^
Load effect (%) = (step duration carrying a load-step duration without carrying a load)/step duration without carrying a load∗ 100					
Initial stairs	.538	14.20 (16.07)	4.44 (7.40)	.127	0.77^b^
Intermediate stairs	.322	18.32 (19.78)	−0.12 (10.94)	.028	1.15^c^
Final stairs	.057	18.61 (12.32)	7.12 (6.73)	.028	1.15^c^
**Full task**	.169	16.89 (13.73)	4.30 (6.84)	.015	1.15^c^

**Notes.**

Values expressed as mean (SD).

* *p*-value of the independent sample Student’s *t*-test or Mann–Whitney *U* test, where appropriate.

a Small effect size (cohen’s *d* < 0.5).

b Medium effect size (cohen’s *d* between 0.5 and 0.8).

c Large effect size (cohen’s *d* > 0.8). Initial stairs: stairs 1, 2 and 3; Intermediate stairs: stairs 4 and 5; Final stairs: stairs 6, 7 and 8. Full task: all eight stairs.

Load effect was significantly different in the two groups. Women with FM showed higher increase in time as a consequence of carrying a load. There were differences in the full task (*p* = .015), the intermediate and the final part of the 10-stairs climbing tasks (*p* = .028). On the other hand, there were no statistically significant differences in the initial stairs. The effect size was medium (>0.5 and <0.8) in the initial stairs and large (>0.8) in the rest of the stairs.

**Figure 1:**
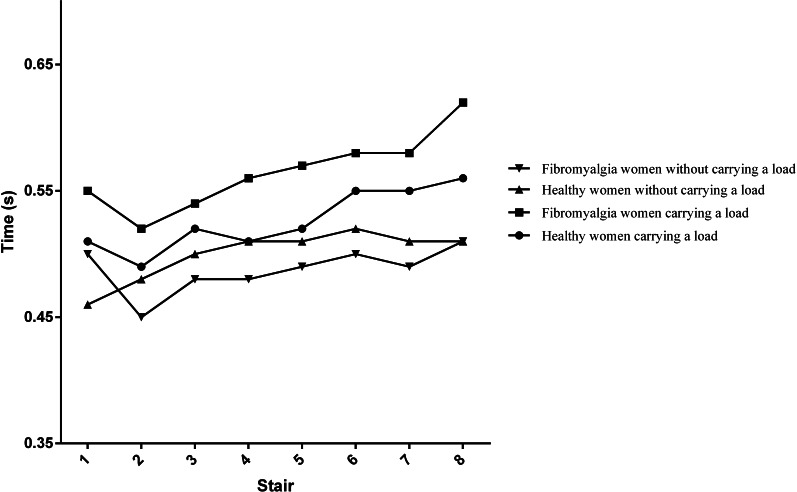
Mean time required for each step.

Figure 1 depicts the evolution of the time required to complete each step. This time was generally on a rising trend in the two groups. The highest rising trend was observed in the group with FM when they were carrying a load of 5 kg in each hand.

## Discussion

The main finding of the current paper was that women with FM altered their motor pattern climbing stairs with loads more than the healthy controls. In more detail, postural sway and the time required to climb stairs were modified differently. Firstly, women with FM significantly increased the trunk tilt when they were carrying a load of 5 kg in each hand compared with the healthy controls. This finding may be extremely relevant as mediolateral stability is closely related to fall risk (Bergland, Jarnlo & Laake, 2003). One previous study evaluated different acceleration parameters in the mediolateral axis during stair ascent using accelerometer data (Wang et al., 2014). They placed an inertial measurement Opal sensor in the centre of the lower back and observed that acceleration parameters in the mediolateral axis were related with physiological risk of falling. Although most of the studies only assess mediolateral acceleration or deviation in static tasks, it is proved that both measures are relevant in dynamic tasks like walking, or stair ascent and descent. The high risk of fall and the serious consequences of falling in stair-climbing make it necessary to assess the balance stability of populations with altered balance, in order to design specific treatments. In this regard, different treatments may be effective in improving the aforementioned mediolateral sway, like whole-body vibration (Sanudo et al., 2013; Sanudo et al., 2012), physical exercise (Jeon et al., 2014), or proprioception training programs (Martinez-Amat et al., 2013). Future studies on the identification of the best treatment in improving mediolateral sway in women with FM are required.

Load effect was calculated as percentage of the increased time in each step as a consequence of the load. The observed slowdown when women with FM were carrying loads might be a consequence of the lack of strength or resistance of the lower limb in women with FM, reported by previous studies (Aparicio et al., 2015; Valkeinen et al., 2008). In fact, previous studies showed that an aquatic program including leg squats improved 10-stair climbing time while carrying loads and leg strength, whereas FM controls progressively worsened their performance and postural balance (Tomas-Carus et al., 2008). Therefore, an implication of the results reported in the current study may be the need of adding resistance and strength training to physical therapies for women with FM.

The step-by-step performance in the stair-climbing tasks is depicted in Fig. 1. The time required to complete each step was on a rising trend for both groups in the two tasks, progressively increasing until the last step. However, this tendency is higher when participants were carrying a load, compared to the weightless task. In addition, this tendency was higher for women with FM. This could indicate that fatigue highly affects as a consequence of the load. This finding is consistent with previous studies that showed the progressive worsening of time climbing 10-stairs, especially while carrying loads (Tomas-Carus et al., 2008; Tomas-Carus et al., 2007). Although pain is a major symptom in FM, no significant linear correlation was found between pain and any of the explored variables. In other words, the slowdown seems to be associated in a greater scale with physical deconditioning than with pain. However, there is a need for further studies testing this hypothesis. Similarly, no significant linear correlation was observed between the explored variables and the question about stair climbing in FIQ and FIQ-R. In the FIQ, the question is “Were you able to climb stairs?” Since all the participants in the current study were able to climb stairs (it was an inclusion criteria), it seems reasonable that correlation was not significant. However, the question in the FIQ-R is “how much your fibromyalgia made it difficult to perform each of the following activities during the past 7 days” and significant correlation was expected. This supports that physical fitness tests are more capable to capture differences in the ability of conducting ADL than self-reported questionnaires in women with FM (von Bulow et al., 2015; Waehrens et al., 2012). Further studies should investigate the relation between the perceived ability to climb stairs and the actual performance in a physical test.

All the reported differences could be even higher, based on characteristics of the participants of the current study. The mean age was almost two years higher for healthy controls compared to women suffering from FM. The severity of FM according to the score in FIQ was “moderate,” as it was between 39 and 59 (Bennett et al., 2009a). Finally, the mean impact of FM on the function domain was low (13.39). Therefore, it could be expected that the differences reported in the current study would be higher with a “severe” score in the FIQ.

### Study limitations

The current study encountered several limitations. Firstly, the cross-sectional nature of the study and its relatively small sample size limited the capability to identify the causes of the reported differences between women with and without FM. Therefore, there is a need for larger studies that could explain the causes of the higher slowdown and trunk tilt. Secondly, the current study did not compare directly and statistically the trunk tilt while carrying and without carrying a load because the motor pattern of arms is inherently different due to the action of carrying weight, which add other source of variability. Therefore, a trunk tilt comparison in the two situations would require a specific design and a larger sample size. The third limitation could be due to the selected tasks limited the muscle fatigue as participants were asked to climb only 10 stairs, which is mainly related to non-fully aerobic mechanisms. Future studies focused on the onset of fatigue could also include higher number of stairs. The fourth limitation could be consequence of the dimensions of the stairs, which may vary from stairs in other countries. In this regard, higher steps could result in greater sway given the greater need for hip flexion. Finally, risk of falling is also high during stair descent (Bosse et al., 2012), but the assessment of the impact of FM (using FIQ, FIQ-R or physical tests) is usually conducted during stair climbing. Despite these limitations, the current study contributes significantly to the research focused on the performance of women with FM during the stair ascent.

## Conclusions

Two main conclusions can be obtained from the results of the current study. In the first place, trunk tilt during the stair climbing while carrying a load was significantly higher for women with FM, which could increase the risk of falling. Second, women with FM experienced a slowdown as a consequence of the load. This slowdown was especially observed at the intermediate and the final part of the 10-stairs climbing test. Therefore, there is a need to prevent the loss of leg resistance and strength, in order to reduce the impact of FM on the ability to perform activities of daily living in women with FM.

## Supplemental Information

10.7717/peerj.1656/supp-1 Dataset S1 Dataset

## Additional Information and Declarations

### Data Availability

The following information was supplied regarding data availability: The raw data is supplied as a Dataset S1.
